# Sector Retinitis Pigmentosa: Extending the Molecular Genetics Basis and Elucidating the Natural History

**DOI:** 10.1016/j.ajo.2020.08.004

**Published:** 2021-01

**Authors:** Michalis Georgiou, Parampal S. Grewal, Akshay Narayan, Muath Alser, Naser Ali, Kaoru Fujinami, Andrew R. Webster, Michel Michaelides

**Affiliations:** a Institute of Ophthalmology, University College London, London, United Kingdom; b **UCL** Institute of Ophthalmology, University College London, London, United Kingdom; c Laboratory of Visual Physiology, Division of Vision Research, National Institute of Sensory Organs, National Hospital Organization Tokyo Medical Center, Tokyo, Japan; d Department of Ophthalmology, Keio University School of Medicine, Tokyo, Japan

## Abstract

**Purpose:**

To determine the genetic background of sector retinitis pigmentosa (RP) natural history to better inform patient counseling.

**Design:**

Retrospective case series.

**Methods:**

Review of clinical notes, retinal imaging including color fundus photography (CFP), fundus autofluorescence (FAF), optical coherence tomography (OCT), electrophysiological assessment (ERG), and molecular genetic testing were performed in patients with sector RP from a single tertiary referral center. Main outcomes measured were demographic data, signs and symptoms, visual acuity, molecular genetics; and ERG, FAF, and OCT findings.

**Results:**

Twenty-six molecularly confirmed patients from 23 different families were identified harboring likely disease-causing variants in 9 genes. The modes of inheritance were autosomal recessive (AR, n=6: *USH1C*, n=2; *MYO7A*, n=2; *CDH3*, n=1; *EYS*, n=1), X-linked (XL, n=4: *PRPS1*, n=1; *RPGR*, n=3), and autosomal dominant (AD, n=16: *IMPDH1*, n=3; *RP1*, n=3; *RHO*, n=10), with a mean age of disease onset of 38.5, 30.5, and 39.0 years old, respectively. Five of these genes have not previously been reported to cause sector RP (*PRPS1*, *MYO7A*, *EYS*, *IMPDH1*, and RP1). Inferior and nasal predilection was common across the different genotypes, and patients tended to maintain good central vision. Progression on serial FAF was observed in *RPGR*, *MYO7A*, *CDH23*, *EYS*, *IMPDH1*, *RP1*, and *RHO*-associated sector RP.

**Conclusions:**

The genotypic spectrum of the disease is broader than previously reported. The longitudinal data provided will help to make accurate patient prognoses and counseling as well as inform patients' potential participation in the increasing numbers of trials of novel therapeutics and access to future treatments.

## Highlights

• This is the largest series and longitudinal study in sector retinitis pigmentosa.
• The genotypic spectrum of the disease is broader than previously reported.
• The longitudinal data provided more accurate patient prognosis and counseling.
• The study informed patients' potential participation in the increasing numbers of trials of novel therapeutics and access to future treatments.

Retinitis pigmentosa (RP) is a heterogeneous group of inherited retinal disorders characterized by nyctalopia, visual field defects, and progressive retinal degeneration.^1^ RP can also exist in syndromic forms, such as Usher syndrome and Bardet-Biedl syndrome.^2^ Sector RP and pericentral RP are atypical variants of RP.^3^^,^^4^

Sector RP was first reported in 1937, with limited reports subsequently, despite representing a distinct phenotype. Sector RP is characterized by typical clinical features of RP limited to 1 or 2 fundus quadrants.^5^ It tends to affect inferior and nasal quadrants with corresponding superior visual field defects.^6^ Sector RP has a favorable visual prognosis compared to generalized RP; it has been reported that 82% of cases will retain a visual acuity (VA) of 20/40 or better.^7^ Autosomal dominant (AD), autosomal recessive (AR), and X-linked (XL) modes of inheritance have been reported. There are 6 previously reported disease-causing genes: rhodopsin (AD: *RHO*: Online Mendelian Inheritance in Man [OMIM] entry 180380; n = 70 cases),^4^^,^^6^^,^^8^, ^9^, ^10^, ^11^, ^12^, ^13^, ^14^, ^15^, ^16^, ^17^ usherin (AR: *USH1C*: OMIM 605242; n = 2)^2^; cadherin 23 (AR: *CDH23*, OMIM 605516; n = 1)^18^; retinol dehydrogenase 5 (AR: *RDH5*, OMIM 601617; n = 1)^19^; arrestin (AR: *SAG*, OMIM 181031; n = 1)^20^; and more recently, RP GTPase regulator gene (XL, *RPGR*, OMIM 312610; n = 2).^4^^,^^21^ All previously reported sector RP-causing variants are summarized in Supplemental Table 1. Currently there are ongoing efforts to develop and approve novel therapeutic options for diseases caused by *RPGR*-RP and *RHO*-RP.^22^^,^^23^

There are limited studies focusing on sector RP. Longitudinal natural history studies particularly are limited to case reports. The present study reports the largest series and first longitudinal study in patients with molecularly confirmed sector RP. Although sector RP is believed to be a mild condition compared to generalized RP, there is a need for more robust data to advise on prognosis. This is particularly true in the molecular era and with the development of novel therapeutics.

This study, therefore, investigated genetic and phenotypic variability in a large cohort of patients with molecularly confirmed sector RP seen in a tertiary center and investigated disease natural history and provided valuable information that can better inform patient counseling and prognostication.

## Subjects and Methods

### Patient Identification

All correspondence contained in an electronic clinical database (OpenEyes) at Moorfields Eye Hospital (MEH, London, United Kingdom) was searched for the key words: “sector,” “sectorial,” and “sectoral.” The clinical notes of all identified patients were reviewed to confirm the diagnosis of sector RP. All patients with a confirmed clinical diagnosis were seen in retinal genetics clinics and were evaluated by experienced specialists (A.R.W., M.M.). Patients were identified in the MEH Inherited Eye Disease database for molecular confirmation. Patients were included in this database after informed consent was obtained. This study adhered to the tenets of the Declaration of Helsinki and was approved by the MEH ethics committee.

### Assessments

Medical notes and clinical images were reviewed, including dilated fundoscopy, VA recording, electrophysiological assessment (using electroretinography [ERG]), retinal imaging (including color fundus photography [CFP] [Optos, Marlborough, Massachusetts; and TopCon, Tokyo, Japan]), optical coherence tomography (OCT) (Spectralis, Heidelberg Engineering, Heidelberg, Germany), and fundus autofluorescence (FAF) (Spectralis and Optos). The age of disease onset was defined as the age of the first disease-related symptom(s). Full-field and pattern ERG were performed using gold foil electrodes to incorporate the International Society for Clinical Electrophysiology of Vision standards (ISCEV) in 9 patients.

### Retinal Imaging

The retina was divided into 4 halves, with the center at the fovea. The superior-inferior and nasal-temporal meridians defined the nasal and temporal retina and the superior and inferior retina, respectively (Supplemental Figure 1 [Supplemental Material is available at *www.ajo.com*]). FAF imaging (55-degrees) and CFP were used to evaluate the extent of retinal involvement. The presence of a perimacular ring of increased signal on FAF was also noted. Macular involvement was also investigated by using all available modalities. Interocular symmetry was evaluated qualitatively, taking into account the topology of the affected quadrants and the extent of degeneration. Eyes with differently affected quadrants were defined as asymmetrical.

Foveal total retinal thickness and outer nuclear layer thickness were calculated at baseline and last follow-up. All measurements were made by a single examiner using the digital calipers built into the software (Heidelberg Eye Explorer; Heidelberg Engineering), with a 1-pixel-to-1-μm display with maximum magnification. Foveal total retinal thickness was measured as the distance between the internal limiting membrane and the retinal pigment epithelium. Outer nuclear layer thickness was measured as the distance between the internal limiting membrane and the external limiting membrane, or the distance between the outer plexiform layer and the external limiting membrane in patients without and with foveal hypoplasia, respectively.

### Statistical Methods

Statistical analysis was carried out using SPSS Statistics (Chicago, Illinois). Significance for all statistical tests was set at *P* <.05. The Shapiro-Wilk test was used to test for normality for all variables.

## Results

### Patient Characteristics

A total of 26 patients with molecularly confirmed sector RP were identified from 23 pedigrees, as noted with a pedigree number (Table 1), harboring likely disease-causing variants in 9 genes. Modes of inheritance were X-linked (XL, n = 4: *PRPS1,* n = 1; and *RPGR,* n = 3); autosomal recessive (AR, n = 6: *USH1C,* n = 2; *MYO7A,* n = 2; *CDH23,* n = 1; and *EYS*, n = 1); and autosomal dominant (AD, n = 16: *IMPDH1,* n = 3; *RP1,* n = 3; and *RHO,* n = 10). Five of these genes have not previously been reported to cause sector RP (*PRPS1*, *MYO7A*, *EYS*, *IMPDH1*, and *RP1*). Molecular genetics, sex, and family history of all patients are presented in the Table 1. The clinical presentation, including the age of disease onset of each genotype, are presented individually below. The 2 siblings carrying *USH1C* variants and sector RP were previously described in detail in the study by Saihan and associates,^2^ and were excluded from OCT, FAF, and further individual analysis.

**Table 1:** Demographics and Genetics

Patient ID	Pedigree	Genetic ID	Gene	Sex	Family History	Variant 1		Variant 2	
						Nucleotide Change	Amino acid Change/Effect	Nucleotide Change	Amino Acid Change/Effect
X-linked sector retinitis pigmentosa									
P1	20175	35460	*PRPS1*	F	Y	c.47C>T	p.Ser16Phe		
P2	1737	21404	*RPGR*	F	Y	c.1239_1243delAGAGA	p.(Glu414Glyfs∗37)		
P3	5345	31560	*RPGR*	M	N	c.3092delA	p.(Glu1031Glyfs∗58)		
P4	4297	26063	*RPGR*	M	Y	c.485_486delTT	p.Phe162Tyrfs∗4		
Autosomal recessive sector retinitis pigmentosa									
P5	16975	26022	*USH1C*	M	Y	c.308 G>A	p.Arg103His	c.2227-1G>T	p.?
P6	16975	30346	*USH1C*	F	Y	c.308 G>A	p.Arg103His	c.2227-1G>T	p.?
P7	20699	31899	*MYO7A*	F	N	c.3476G>T	p.Gly1159Val	c.3728C>T	p.Pro1243Leu
P8	19131	29686	*MYO7A*	M	N	c.22dupG	p.Asp8Glyfs∗34	c.6551C>T	p.Thr2184Met
P9	21894	33976	*CDH23*	M	N	c.5237G>A	p.Arg1746Gln	c.9278+2T>G	p.?
P10	22692	34950	*EYS*	F	Y	c.6794delC	p.Pro2265Glnfs∗	c.8278C>T	p.Arg2760Cys
Autosomal dominant sector retinitis pigmentosa									
P11	20700	31900	*IMPDH1*	M	Y	c.1074+6_1074+7delGCinsTT	p.?		
P12	18732	28954	*IMPDH1*	F	Y	c.968A>G	p.Lys323Arg		
P13	24034	37043	*IMPDH1*	F	N	c.1603A>G	p.Lys535Glu		
P14	3650	2322	*RP1*	F	y	c.2172_2185del	p.Ile725Argfs∗6		
P15	21079	32583	*RP1*	F	Y	c.2029C>T	p.Arg667∗		
P16	18591	28719	*RP1*	F	Y	c.2206dupA	p.Thr736Asnfs∗4		
P17	16765	23876	*RHO*	F	Y	c.165C>A	p.Asn55Lys		
P18	2482	1535	*RHO*	M	Y	c.937-1G>T	p.?		
P19	1379	4976	*RHO*	F	Y	c.410G>T	p.Met39Arg		
P20	1379	22517	*RHO*	F	Y	c.410G>T	p.Met39Arg		
P21	3509	21686	*RHO*	F	y	c.568G>A	p.Asp190Asn		
P22	3509	9533	*RHO*	M	y	c.568G>A	p.Asp190Asn		
P23	1895	9592	*RHO*	F	Y	c.316G>A	p.Gly106Arg		
P24	3492	10471	*RHO*	F	N	N	c.467C>G	p.Thr58Arg	
P25	2554	9924	*RHO*	F	Y	N	c.568G>A	p.Asp190Asn	
P26	19172	29744	*RHO*	M	y	N	c.116T>G	p.Met39Arg	

Gene (transcript GenBank accession ID): *CDH23* (NM_022124.6); *EYS* (NM_001142800.1); *IMPDH1* (NM_000883.4); *MYO7A* (NM_000260); *PRPS1* (NM_002764.4); *RHO* (NM_000539.3); *RP1* (NM_006269.2); *RPGR* (NM_001034853.2); *USH1C* (NM_153676.4).

### Visual Acuity and Disease Symmetry

**Figure 1:**
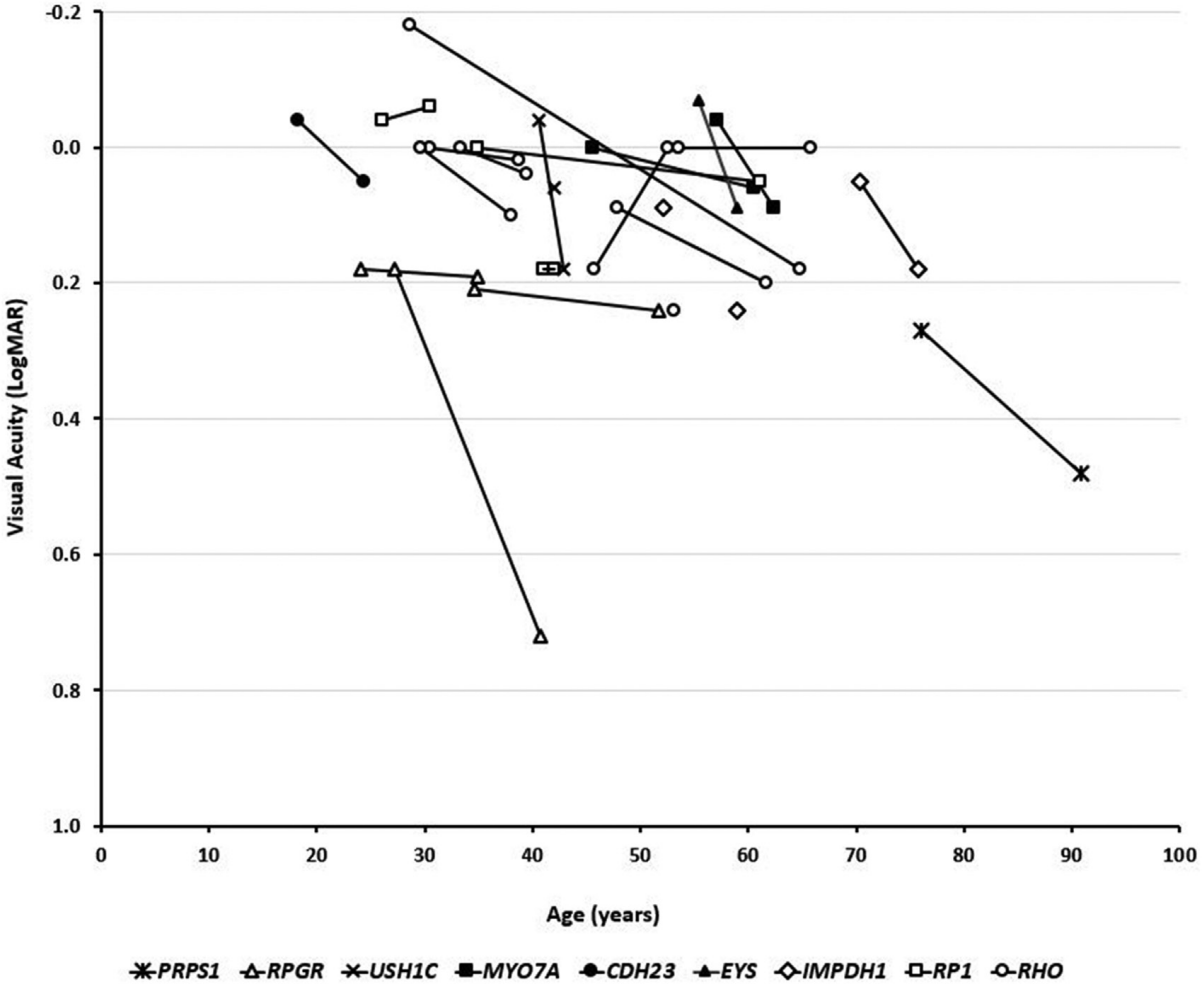
Visual acuity graphical representation. Twenty-four patients had available visual acuity data; 20 patients were longitudinally assessed and were followed for a mean of 10.6 years (range: 2.3-36.1 years). The graph represents visual acuity at baseline and follow-up (where available), over age. Each genotype is presented with a different marker.

VA was available for 24 of the 26 patients. Of the 24 patients, 2 had reduced VA in 1 eye unrelated to retinal degeneration (P1: left eye showed closed angle glaucoma and cataract; P19: right eye showed amblyopia) and were excluded from VA interocular comparison. The mean VA at baseline examination (including mean age: 42.8; range: 18.3-76.1 years) for the right and left eyes was 0.035 and 0.061 LogMAR, respectively. Right and left eye VA were similar (*P* = .203; t = −1.31; *df* = 21). Twenty patients had longitudinal VA assessment, with a mean follow-up of 10.6 years (range: 2.3-36.1 years). Final mean VA measurements were 0.06 and 0.10 LogMAR for right and left eyes, respectively. VA was statistically significantly worse at follow-up (*P* = .007; t = −3.05; *df* = 19). Figure 1 presents the VA for all patients over time, with genotypical details.

**Figure 2:**
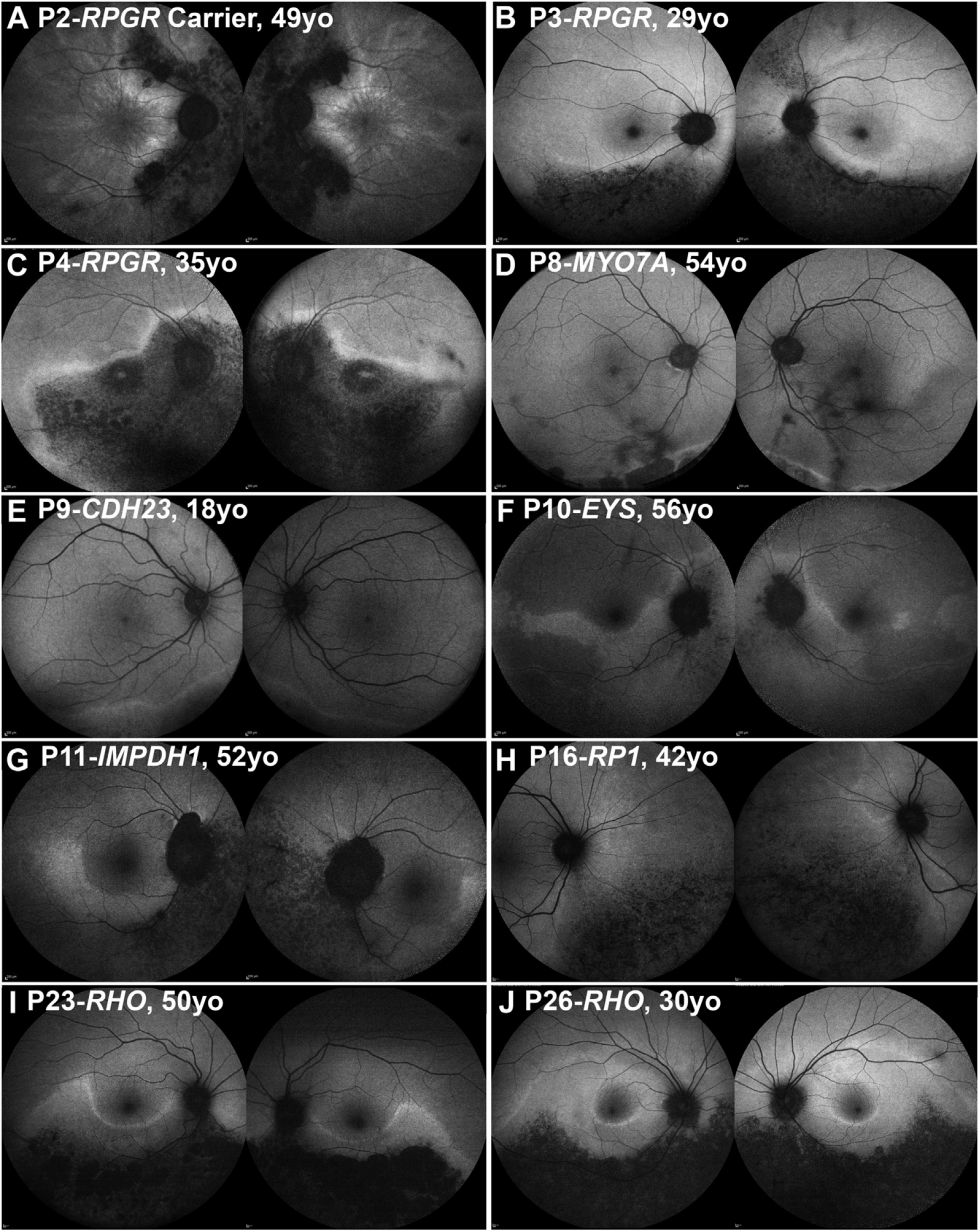
Disease symmetry. Fundus autofluorescence imaging of the right and left eyes of 10 patients (A-J), with sector retinitis pigmentosa. The disease was symmetrical in all cases, except for P3 (B). The age and the genotype of everyone is noted in the figure.

**Figure 3:**
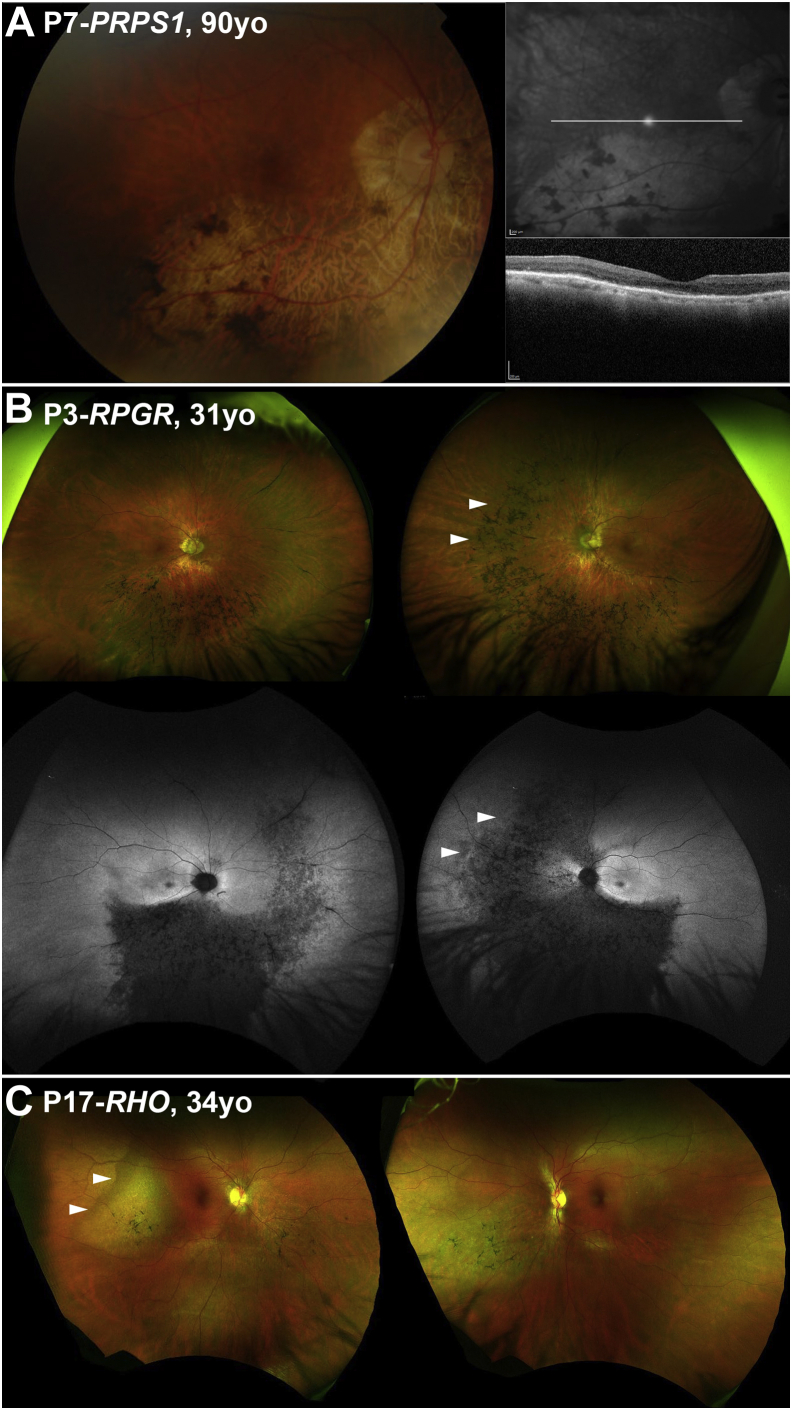
Examples of disease asymmetry. (A) *PRPS1* is known to cause asymmetrical disease in women. Multimodal imaging data were available only from the right eye precluding interocular comparison. (Left) Color fundus photograph. (Right) Near-infrared imaging of the right eye with the white line marking the location of the transfoveal optical coherence tomography scan presented below. (B and C) Examples of interocular asymmetry. (B) P3 has more advanced disease nasally in the left eye (white arrow heads), which are better visualized with fundus autofluorescence imaging (second line). (C) Color fundus photograph of P17, with more advanced disease temporal to the fovea in the right eye (white arrow heads).

In all patients evaluated for interocular symmetry (n = 25), the disease was symmetrical between eyes in 23 patients (92%) in terms of available CFP, OCT, and FAF results. Figure 2 presents FAF examples of interocular symmetry for all genotypes, except those carrying the *PRPS1* gene. Although *PRPS1* is known to cause asymmetrical disease in females,^24^ imaging data were available only from the right eye (due to dense left cataract and prior left angle closure) precluding interocular comparison (Figure 3A). The 2 other patients (P3-*RPGR* and P17-*RHO*) with intraocular differences in presentation were noted only on wide-field imaging (Figure 3, B and C).

### OCT Quantitative and Qualitative Assessments

**Figure 4:**
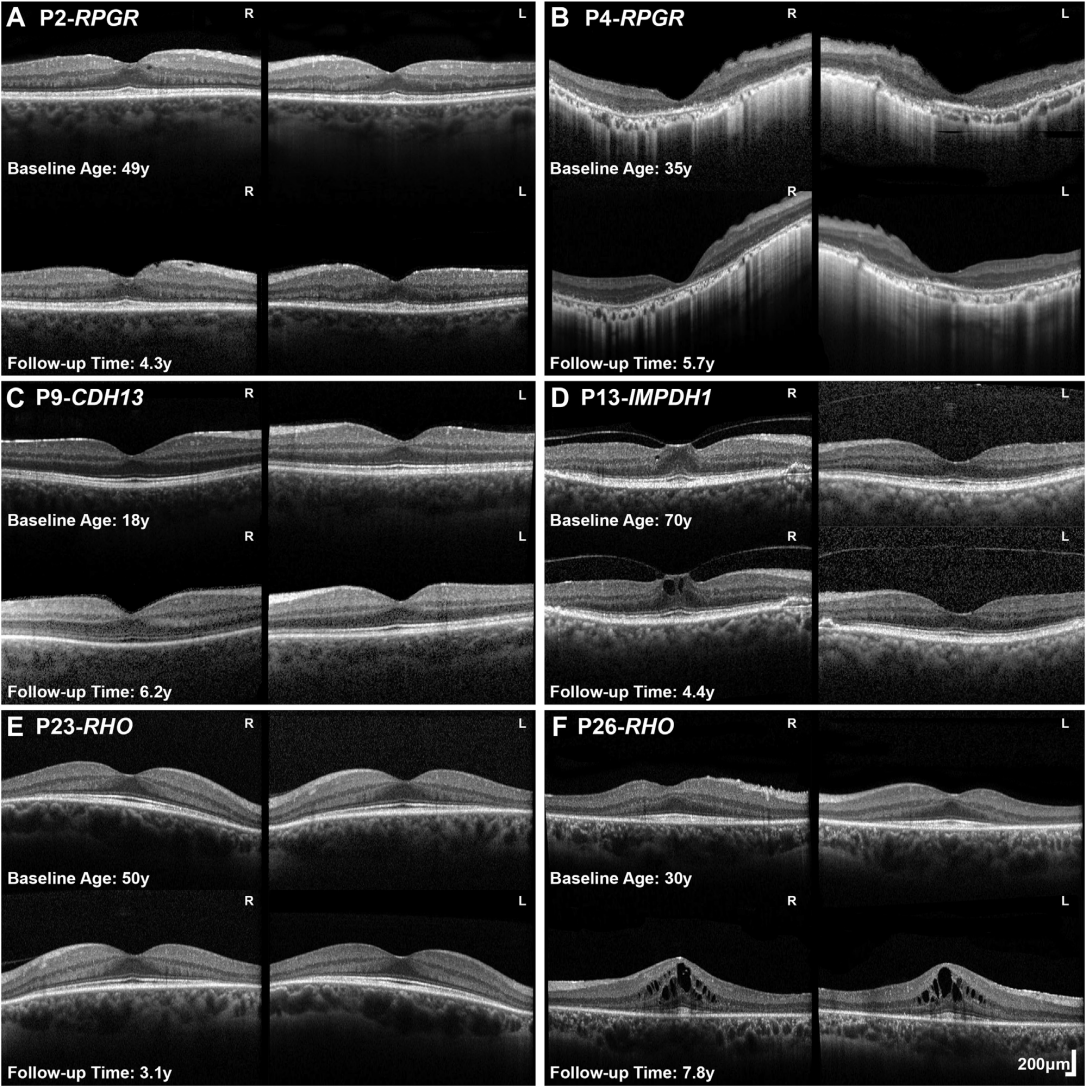
Optical coherence tomography (OCT) in sector RP. (A-F) Transfoveal horizontal OCT scans of 6 patients with sector RP of both eyes at baseline and follow-up. Genotype, age at baseline and follow-up time are noted in the figure. (A, C, E) Patients had no foveal involvement and stable disease. (B) P4 had foveal involvement with no evidence of progression over follow-up. (D) P13 had vitreomacular traction (P13, stage 1 in the right eye). (A) P2, (D) P13, and (F) P26, had various degrees of cystoid macular edema with (D) likely being secondary to traction. L = left eye; R = right eye; RP = retinitis pigmentosa; y = years.

OCT imaging was performed at least once in 18 patients (mean age: 46 years; range: 18-90 years old). Mean foveal thickness was 156 μm and 152 μm for right and left eyes, respectively. Mean outer nuclear layer thickness was 124 μm and 120 μm for right and left eyes, respectively. Previously reported mean ± SD from unaffected controls for outer nuclear layer thickness are 112.9 ± 15.2 (right eye) and 112.1 ± 13.9 μm (left eye).^25^ Patient 4 (P4; 5.6%) had macular involvement with loss of the macular ellipsoid zone (Figure 4, B).

Thirteen patients had serial OCT assessment at a mean final follow-up of 5.6 years (range: 4.4-8.1 years). Mean foveal thickness was 154 μm and 152 μm for right and left eyes, respectively. Mean outer nuclear layer thickness was 117 μm and 112 μm for right and left eyes, respectively. Examples of OCT interocular symmetry and disease progression are presented in Figure 4. Cystoid macular edema (CME) was observed in 4 patients (P2-*RPGR*) (Figure 4, A), P11-*IMPDH1*, P13-*IMPDH1* (Figure 4, D), and P26-*RHO* (Figure 4, F), with 1 having XL (P2) and 3 having AD modes (P11, P13, P26) inheritance mode. Three patients had epiretinal membrane (P2, P12, and P14) (Figure 4, A). One patient had focal vitreomacular traction (VMT; P13, Stage 1) (Figure 4, D).

### Disease Localization and Evidence of Progression

**Figure 5:**
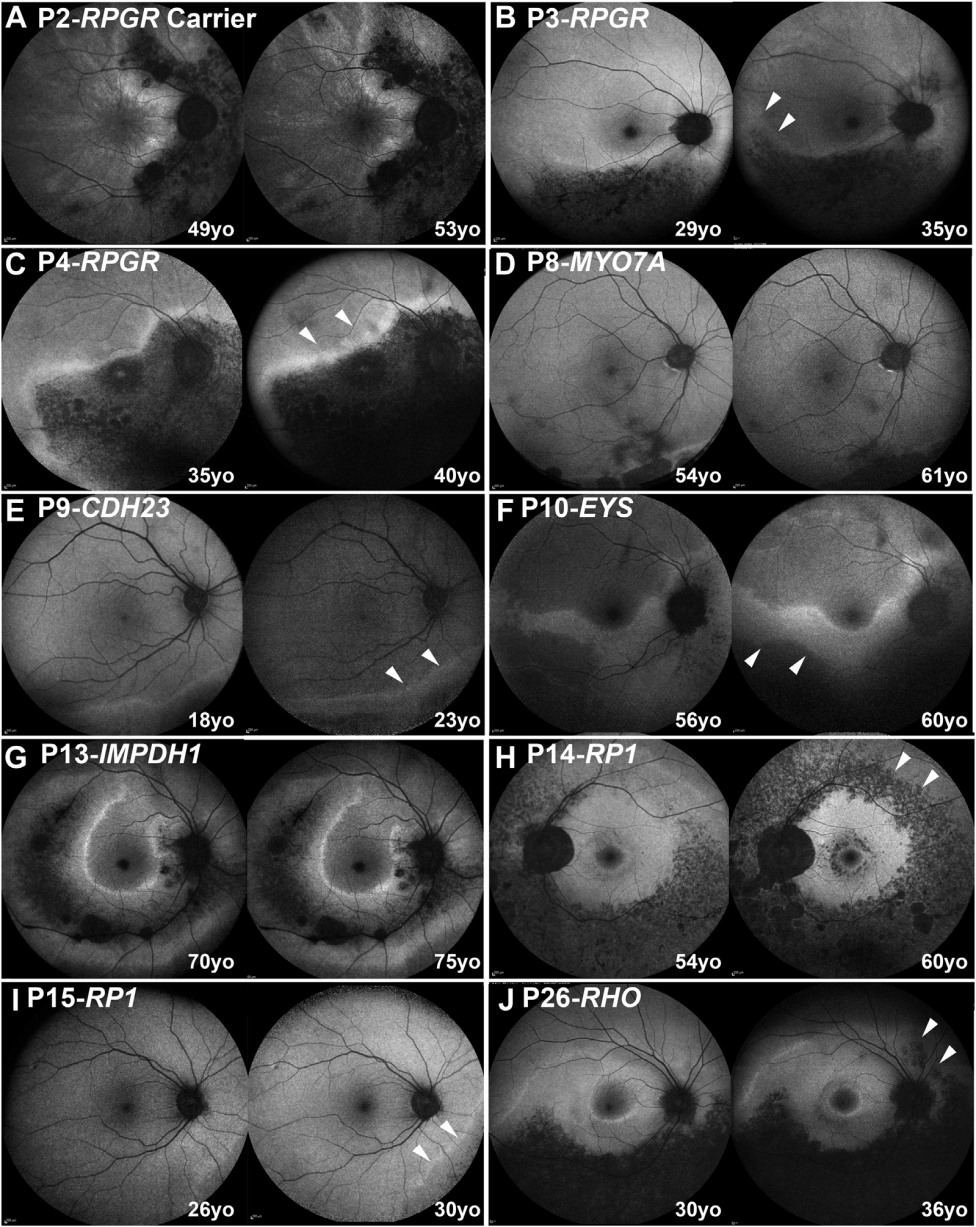
Disease progression. Fundus autofluorescence imaging of the right or left eyes of 10 patients (A-J), with sector retinitis pigmentosa at baseline and follow-up. The genotype of the patient and the age at baseline and follow-up is noted in the figure. The white arrow heads mark the areas of progression in 7 patients. Any progression noted was small in area and extent, with most likely no or limited clinical impact. yo = years old.

FAF imaging was used for localization of the disease and investigation of structural progression due to the peripheral nature of the disease and the wider field of view than with OCT. Eighteen patients had 55-degree FAF imaging (mean age: 46 years old, range: 18.3-70.4 years old). Fifteen patients (83.3%) had peripapillary atrophic changes (Figure 5, A through D and F through J). The 3 patients without peripapillary changes were P7, P9 (Figure 5, E) and P16, with *MYO7A*, *CDH23*, and RP1 genotypes, respectively. A common finding was a hyperautofluorescent rim, bordering areas of affected and healthy retina (n = 13, 72.2%) (Figure 2, Figure 5). A perifoveal ring of increased signal was present in 2 subjects (11.1%; P14-RP1) (Figure 5, H) and P26-*RHO* (Figure 5, J). Both patients had hyperautofluorescent rings and rims. Foveal involvement was observed only in one subject (P4-*RPGR*) (Figure 5, C). Disease localization was noted: *i)* nasal (n = 1, 5.6%, P2-*RPGR* carrier) (Figure 5, A); *ii)* inferior (n = 6, 33.3%); *iii)* inferior and nasal (n = 9; 50%); and *iv)* inferior, temporal, and nasal (n = 2; 11.2%; P13-*IMPDH1* and P14-RP1). Disease localization for each individual patient is presented in Supplemental Table 2.

Sixteen patients had available follow-up imaging (mean follow-up time, 5 years; range: 1-9.1 years). Seven of the 16 patients (43.8%) showed changes in the FAF signal. Figure 5 presents all 6 patients with evidence of progression. Changes were peripheral and small in area and most likely of limited clinical significance. Only 1 patient showed extension of the disease to the superior retina (P14-*RP1*) having a more “typical” RP presentation, with mid-peripheral changes in all quadrants (Figure 5, H). The mean follow-up of the patients with structural changes was 5.6 years (range: 4-7.8 years).

### X-Linked Sector RP

#### *PRPS1* (n = 1)

Patient P1 was diagnosed at age 45 and presented with reduced vision. Vision in the left eye was perception of light and without clear visual axis due to previous angle closure glaucoma. Inferior atrophy with pigmentary changes were documented on fundoscopy. The VA in the right eye was 0.27 LogMAR at 76 years of age and only mildly deteriorated to 0.48 LogMAR at 91 years of age. Although no FAF imaging was available for disease localization, CFP documented the disease in the inferior retina (Figure 3, A).

#### *RPGR* (n = 3)

Three patients harbored *RPGR* ORF15 variants. One female carrier (P2) had an onset of disease at 32 years old, presenting with increased difficulty with night vision. VA was 0.24 and 0.18 LogMAR at 35 years of age and remained relatively stable at 0.18 and 0.3 LogMAR after 16 years of follow-up for right and left eye, respectively. The two affected men had an earlier age of onset, at 4 years (P3) and 10 years (P4), with the latter diagnosed at asymptomatic screening by an optometrist and the former having reduced color vision. VA for P3 at 18 years of age was 0.18 LogMAR in both eyes. At 37 years old, patient P4 had a baseline VA of 0.18 LogMAR in both eyes and, over 13.5 years of follow-up, deteriorated to 0.92 and 0.52 LogMAR in the right and left eyes, respectively. Greater disease progression and VA deterioration was observed in the right eye due to foveal involvement (Figure 5, C). All 3 patients showed involvement of the inferior and nasal retina on FAF results (Figure 5, A to C). The affected female also had a tapetal-like reflex, a common finding among *RPGR* carriers,^26^ visible both on fundoscopy and FAF (Figure 2, A). Patient P2 underwent ERG testing at 34 years of age, which showed generalized retinal dysfunction, affecting both the cone and the rod systems, with macular involvement. Rod-specific ERG was precluded by blink artifacts. Patient P3 had ERG testing at 29 years of age, which showed generalized retinal dysfunction affecting more of the rod than the cone system, with paracentral macular involvement. Interestingly, an affected male cousin of patient P3 had a symmetrical, nonsectorial phenotype of cone-rod dystrophy, despite harboring the same *RPGR* variant.

### Autosomal Recessive Sector RP

#### *MYO7A* (n = 2)

**Figure 6:**
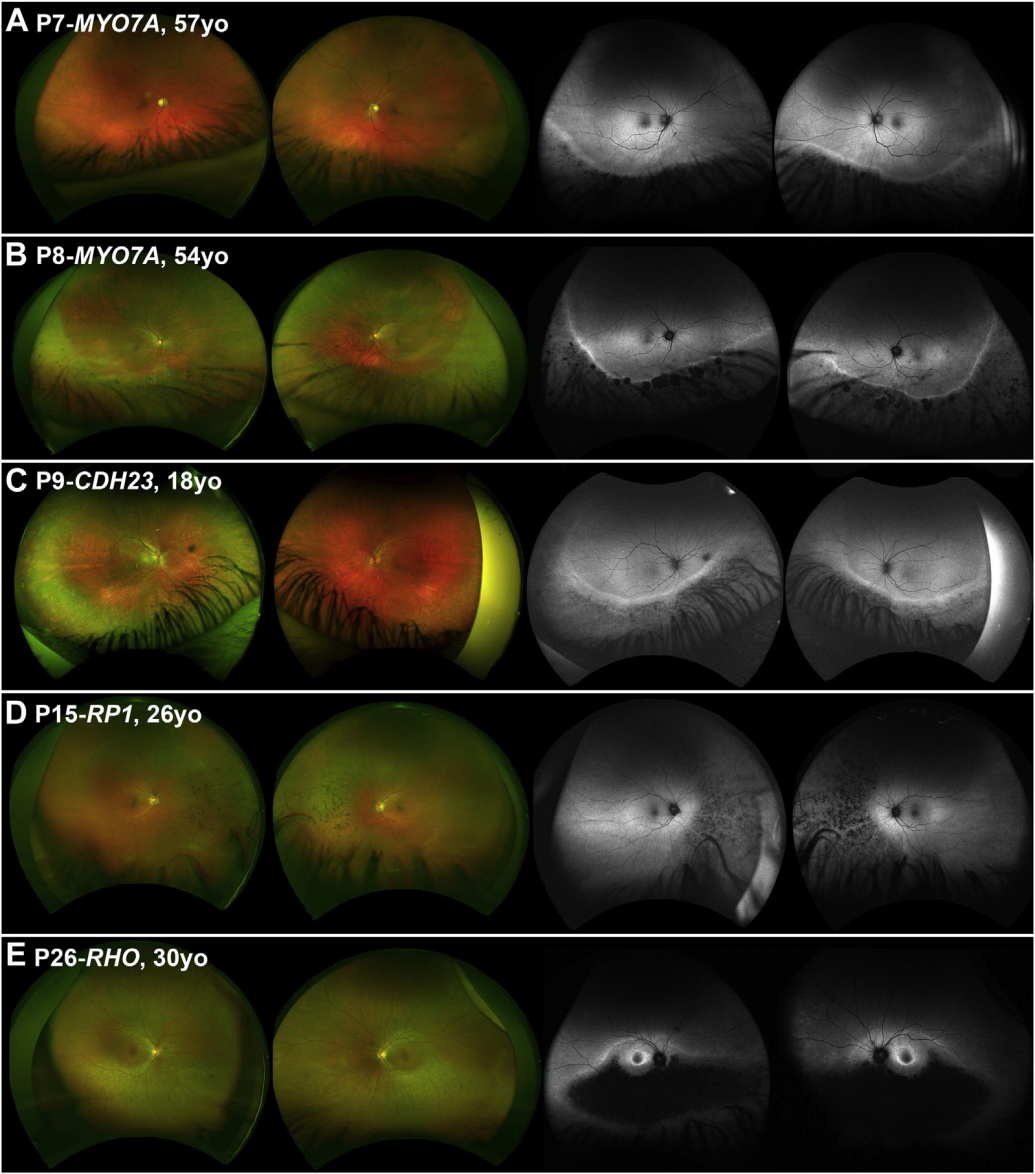
Wide-field imaging in sector retinitis pigmentosa (RP). (Left) Color fundus photographs of the right and left eyes of 5 patients (A-E) with sector RP. (Right) Corresponding fundus autofluorescence (FAF) images. Genotype and age of each patient is presented in the figure. (A-C) Disease is extending well below the arcades and was thereby not possible to fully assess with conventional 55-degree FAF (see Figure 2).

Patients P7 and P8, who were from 2 independent pedigrees and both severely hearing impaired, were referred for evaluation of pigmentary changes in the inferior retina after routine optometry assessment (Figure 6, A and B), without any associated visual complaint, at 53 and 45 years old, respectively. Superior field defects were noted in both patients. VA for P7 was 0 and −0.08 LogMAR at age 57, and 0.0 and 0.16 LogMAR at age 62 years for right and left eyes, respectively. VA for patient P8 was 0.0 LogMAR for both eyes at age 46, and 0.12 and 0.0 LogMAR for right and left eyes, respectively, after 15 years of follow-up. Patient P7 had normal pattern and full-field ERG results at 58 years of age, and P8 had a normal ERG pattern and mildly subnormal full-field ERG with normal peak times at 44 years of age.

#### *CDH23* (n = 1)

Patient P9 had Usher syndrome Type 1, and he was referred at 18 years of age for evaluation (Figure 6, C). He had bilateral cochlear implants. VA was 0.0 and −0.08 LogMAR at 18 years of age, and 0.04 and 0.06 after 6 years of follow-up for the right and left eye, respectively. Previous ERG testing at 7 years of age showed normal responses from both eyes, which deteriorated after 8 years of follow-up both for the rod and to a lesser extent the cone system.

#### *EYS* (n = 1)

At 54 years of age, patient P10 was noted by her optometrist to have bilateral superotemporal visual field defects and was referred for evaluation. VA was −0.14 and 0.0 LogMAR at 55 years of age, and 0.0 and 0.18 after 4 years of follow-up in the right and the left eye, respectively. Vessel attenuation, pale discs, and inferior retinal pigment epithelial granularity were observed on fundoscopy and remained relatively stable during follow-up and was visible on FAF (Figure 5, F). ERG at 56 years of age showed a reduction in rod and cone amplitudes, with no peak-time shift.

### Autosomal Dominant Sector RP

#### *IMPDH1* (n = 3)

The mean age of presentation was 56.3 years old (range: 52.1-70.4 years). Patients P11 and P13 were asymptomatic and incidentally noted to have retinal changes. P12 presented with peripheral field constriction and difficulty focusing. The mean presenting VA was 0.06 logMAR (range: 0.00-0.18 logMAR). Patient P12 had superior visual field constriction to 20-degrees. P11 was the only patient who had an ERG which demonstrated generalized retinal dysfunction affecting the rod more than the cone system. None of the patients had foveal involvement or progression on FAF (Figure 2, Figure 5, G).

#### *RP1* (n = 3)

The mean age of presentation was 33.1 years old (range: 26.0-41.1 years). The mean presenting VA was 0.06 logMAR (range: 0.00-0.18 logMAR). On ERG, patient P14 had generalized dysfunction of the rod system, and P16 had generalized retinal dysfunction of both the rod and the cone systems. Two of the patients showed evidence of mild progression on FAF (Figure 5, H and I).

#### *RHO* (n = 10)

Patients carrying *RHO* variants had a mean age of 52.2 years old at presentation (range: 28.6-61.7 years). Four patients presented with nyctalopia, 5 presented with peripheral visual symptoms, and the last patient was asymptomatic. The mean presenting VA was 0.03 logMAR (range: 0.00-0.24 logMAR). None of the patients had foveal involvement, and only 1 had evidence of mild progression on FAF (Figure 5, J).

## Discussion

This study investigated the genetic and phenotypic variation in the largest cohort of patients who were molecularly confirmed with sector RP in the medical literature. Analysis identified 9 genes as causative for the disease, of which 5 were not previously implicated (*PRPS1*, *MYO7A*, *EYS*, *IMPDH1*, and *RP1*). The study provided data for disease natural history and information that can help inform patient counseling and prognosis for each individual genotype.

Sector RP has a favorable visual prognosis compared to “typical” RP. It has been reported that 82% of patients retain a VA of 0.3 LogMAR or better (n = 17, not molecularly confirmed).^27^ In the present cohort, 24 patients (24 of 26; 83.3%) had VA better than 0.3 LogMAR (Figure 1). Coussa and associates^4^ reported that those with sector RP due to *RHO* (n = 9) retained relatively good central vision (better than 0.18 LogMAR). In agreement with that study, 7 of 8 patients (87.5%) of patients in the present study with *RHO* variants had VA equal to or better than 0.18 LogMAR. In the present cohort, relative structural and functional stability were observed across all genotypes during follow-up (Figure 1, Figure 5). The present authors recently reported 105 families affected with *RHO*-associated disease in the authors' genetic databse^28^; the current report found 8 of them were associated with sector RP (7.6%). A predisposition for inferior and/or nasal retinal involvement has been reported for sector RP due to *RHO*^6^ and that was also observed in all cases in the current report. There are ongoing human clinical trials of antisense oligonucleotide therapy and hydroxychloroquine (AURORA [Study to Evaluate the Safety and Tolerability of QR-1123 in Subjects With Autosomal Dominant Retinitis Pigmentosa Due to the P23H Mutation in the *RHO* Gene; NCT04123626]; and *RHO* [Oral Hydroxychloroquine {HCQ} for Retinitis Pigmentosa Caused by P23H-Rhodopsin; NCT04120883]), respectively, for patients harboring the *RHO* variant P23H. This variant has also been reported in sector RP.^6^^,^^8^ None of the 8 families in the present study harbored P23H; however, this variant is common in the United States. Overall, the present data support the fact that most patients with sector RP, albeit with a degree of variability depending on the genotype, can be advised of a good prognosis, with serial monitoring for progression with wide-field imaging and for secondary complications with OCT (eg, CME) (Figure 5, F).

*RPGR*-associated sector RP appears to have features that are distinct from other genotypes and a worse prognosis. Two patients are reported to carry *RPGR* variants and sectoral disease (Table 1): 1 of those patients had impaired central vision with asymmetry between the eyes (0.48 and 1.3 LogMAR),^4^ and the other was described as having cone-rod dystrophy (rather than RP-related rod-cone dystrophy) and sectoral disease, also presenting with impaired central vision.^21^ *RPGR* patients (n = 3) tended to have worse VA for their age in the cohort (Figure 1). Asymmetry in VA was noted in patient P4 over the follow-up (0.4 LogMAR interocular differences) and can be attributed to foveal involvement, where small structural changes can have a more dramatic effect on VA. Both of the aforementioned patients^4^ (reported recently from 2 independent centers), and the present patient P3 had a small deletion leading to a frameshift at the same location (c.3092). It should also be highlighted that P2 was an *RPGR* carrier and, to the best of the authors' knowledge, was the first reported carrier of sector RP (Figure 2, A). In all the patients reported with *RPGR* variants, the changes were primarily nasal and peripapillary (Figure 2, A through C). Trials of gene augmentation therapy are already underway for *RPGR*-associated RP (NCT03252847, NCT03116113, and NCT03316560).

Usher syndrome is genetically and phenotypically heterogeneous, and the current report extends the phenotypic spectrum of the retinal manifestations of the causative genes. The *USH1C* gene causes Usher type 1C (*USH1C*, OMIM 276904) and has previously been associated with sector RP (patients P5 and P6 in the current study).^2^ *MYO7A* and *CDH23* are causes of Usher syndrome 1B (USH1B) (OMIM 276900) and -1D (OMIM 601027), respectively. Patients P7 and P8 with *MYO7A* had severe congenital hearing loss, could articulate, and wore hearing aids, suggesting some useful hearing, which is not typical in USH1B. In keeping with the milder hearing deficit, retinal disease was also mild and limited to the inferior retina. Both patients have minimal ERG abnormality, in contrast to the usually undetectable responses in *MYO7A*-associated USH1B (88.6%).^29^ Three of the 4 variants identified in P7 and P8 are missense changes and may represent hypomorphic alleles, thereby allowing residual cochlear function and sector RP. Patient P9 harboring *CDH23* variants was compound heterozygous for a null and a missense variant. Nonsyndromic deafness is associated with *CDH23* missense variants that are presumed to be hypomorphic alleles with sufficient residual activity for retinal function but not for auditory cochlear function. In contrast, null *CDH23*, or a combination of a null allele and a missense in a compound heterozygote, cause USH1D.^30^ The present case is the second case reported in the medical literature where the combination of a null and missense variant led to deafness and sector RP (ie, mild retinal manifestations).^18^

*RP1* encodes a microtubule-associated protein which is thought to be retina-specific, and sequence variants are known to cause AD and AR RP.^31^^,^^32^ Notably, AD *RP1* RP has a relatively mild phenotype, with variants clustered in the large terminal exon 4, as was the case in patients P14-P16 in the present study. Previously reported variants are usually truncating,^33^ as was also the case in the present patients. Variants in other exons can cause a more severe phenotype including early onset retinal degeneration but only when homozygous.^32^ Of note, 2 of the present 3 patients with *RP1* showed some progression on serial FAF; however, none demonstrated foveal involvement. *IMPDH1* encodes inosine-5-prime-monoposphate dehydrogenase, which is responsible for guanine nucleotide biosynthesis. It has been reported to cause AD RP and rarely Leber congenital amaurosis.^34^ *IPMDH1*-RP has been attributed to protein misfolding as opposed to reduced enzymatic activity.^35^ Sector RP due to the *RP1* and *IMPDH1* genes represent a milder phenotype, with no definite genotype-phenotype association evident, suggesting other modifying molecular or environmental factors.

Light exposure has been implicated, given the inferior retinal predilection of sector RP. Light deprivation reduces retinal degeneration in *RHO*-related RP animal models.^36^ Animal models exhibiting rhodopsin glycosylation deficiency are vulnerable to light-related retinal degeneration.^37^^,^^38^ This may be clinically relevant as occupationally related high sunlight exposure may lead to a more severe phenotype, potentially (at least in part) explaining the commonly observed intrafamilial phenotypic variability in *RHO*-RP.^39^ Although the underlying molecular mechanism remains unclear, it is interesting that the inferior predilection was common among the 9 causative genes identified herein. Speculation of a common or down-stream mechanism of light-induced damage is possible. Gradients of gene expression in the retina that are not normally clinically manifest may also predispose the retinal quadrants differently. Given the current evidence, it is reasonable to also advise patients with sector RP to use protection and minimize exposure to light.

The present series highlights the fact that the genotypic spectrum is broader. However, as physicians in a single tertiary referral center, it is difficult to draw conclusions for disease prevalence within the general population, as well as about the prevalence of each gene in patients with sector RP, because mild cases or cases with dominant inheritance may lack molecular confirmation. This study was retrospective, and as a result, not all data were available for all patients. Further evaluation and quantification of the ERG parameters and use of wide-field imaging longitudinally will be of value to monitor disease progression.

This study presented the largest cohort of patients molecularly confirmed with sector RP. The genotypic spectrum of the disease is broader than previously reported, with 5 novel genes causing sector RP identified. The longitudinal data provided will be valuable to better inform patient prognosis and counseling.

## References

[1] Verbakel S.K., van Huet R.A.C., Boon C.J.F. (2018). Non-syndromic retinitis pigmentosa. Prog Retin Eye Res.

[2] Saihan Z., Stabej Ple Q., Robson A.G. (2011). Mutations in the USH1C gene associated with sector retinitis pigmentosa and hearing loss. Retina.

[3] Karali M., Testa F., Brunetti-Pierri R. (2019). Clinical and genetic analysis of a European cohort with pericentral retinitis pigmentosa. Int J Mol Sci.

[4] Coussa R.G., Basali D., Maeda A. (2019). Sector retinitis pigmentosa: Report of ten cases and a review of the literature. Mol Vis.

[5] Bietti G. (1937). Su alcone forme atipiche o rare di degenerazione retinica (degenerazioni tappetoretiniche e quadri morbosi similari). Boll Oculist.

[6] Stone E.M., Kimura A.E., Nichols B.E. (1991). Regional distribution of retinal degeneration in patients with the proline to histidine mutation in codon 23 of the rhodopsin gene. Ophthalmology.

[7] Grover S., Fishman G.A., Anderson R.J. (1999). Visual acuity impairment in patients with retinitis pigmentosa at age 45 years or older. Ophthalmology.

[8] Heckenlively J.R., Rodriguez J.A., Daiger S.P. (1991). Autosomal dominant sectoral retinitis pigmentosa. Two families with transversion mutation in codon 23 of rhodopsin. Arch Ophthalmol.

[9] Fishman G.A., Stone E.M., Gilbert L.D., Sheffield V.C. (1992). Ocular findings associated with a rhodopsin gene codon 106 mutation. Glycine-to-arginine change in autosomal dominant retinitis pigmentosa. Arch Ophthalmol.

[10] Moore A.T., Fitzke F.W., Kemp C.M. (1992). Abnormal dark adaptation kinetics in autosomal dominant sector retinitis pigmentosa due to rod opsin mutation. Br J Ophthalmol.

[11] Kranich H., Bartkowski S., Denton M.J. (1993). Autosomal dominant “sector” retinitis pigmentosa due to a point mutation predicting an Asn-15-Ser substitution of rhodopsin. Hum Mol Genet.

[12] Rivera-De la Parra D., Cabral-Macias J., Matias-Florentino M. (2013). Rhodopsin p.N78I dominant mutation causing sectorial retinitis pigmentosa in a pedigree with intrafamilial clinical heterogeneity. Gene.

[13] Budu, Matsumoto M., Hayasaka S. (2000). Rhodopsin gene codon 106 mutation (Gly-to-Arg) in a Japanese family with autosomal dominant retinitis pigmentosa. Jpn J Ophthalmol.

[14] Ramon E., Cordomi A., Aguila M. (2014). Differential light-induced responses in sectorial inherited retinal degeneration. J Biol Chem.

[15] Napier M.L., Durga D., Wolsley C.J. (2015). Mutational analysis of the rhodopsin gene in sector retinitis pigmentosa. Ophthalmic Genet.

[16] Shah S.P., Wong F., Sharp D.M., Vincent A.L. (2014). A novel rhodopsin point mutation, proline-170-histidine, associated with sectoral retinitis pigmentosa. Ophthalmic Genet.

[17] Xiao T., Xu K., Zhang X. (2018). Sector Retinitis Pigmentosa caused by mutations of the RHO gene. Eye (Lond).

[18] Branson S.V., McClintic J.I., Stamper T.H. (2016). Sector retinitis pigmentosa associated with novel compound heterozygous mutations of CDH23. Ophthalmic Surg Lasers Imaging Retina.

[19] Sato M., Oshika T., Kaji Y., Nose H. (2004). A novel homozygous Gly107Arg mutation in the RDH5 gene in a Japanese patient with fundus albipunctatus with sectorial retinitis pigmentosa. Ophthalmic Res.

[20] Nakamachi Y., Nakamura M., Fujii S. (1998). Oguchi disease with sectoral retinitis pigmentosa harboring adenine deletion at position 1147 in the arrestin gene. Am J Ophthalmol.

[21] Nguyen X.T., Talib M., van Schooneveld M.J. (2020). RPGR-associated dystrophies: clinical, genetic, and histopathological features. Int J Mol Sci.

[22] Tee J.J., Smith A.J., Hardcastle A.J., Michaelides M. (2016). RPGR-associated retinopathy: clinical features, molecular genetics, animal models and therapeutic options. Br J Ophthalmol.

[23] Sudharsan R., Beltran W.A. (2019). Progress in gene therapy for rhodopsin autosomal dominant retinitis pigmentosa. Adv Exp Med Biol.

[24] Fiorentino A., Fujinami K., Arno G. (2018). Missense variants in the X-linked gene *PRPS1* cause retinal degeneration in females. Hum Mutat.

[25] Mastey R.R., Gaffney M., Litts K.M. (2019). Assessing the interocular symmetry of foveal outer nuclear layer thickness in achromatopsia. Transl Vis Sci Technol.

[26] Kalitzeos A., Samra R., Kasilian M. (2019). Cellular imaging of the tapetal-like reflex in carriers of RPGR-associated retinopathy. Retina.

[27] Grover S., Fishman G.A., Alexander K.R. (1996). Visual acuity impairment in patients with retinitis pigmentosa. Ophthalmology.

[28] Pontikos N., Arno G., Jurkute N. (2020). Genetic basis of inherited retinal disease in a molecularly characterised cohort of over 3000 families from the United Kingdom. Ophthalmology.

[29] Khateb S., Mohand-Said S., Nassisi M. (2019). Phenotypic characteristics of cone-rod dystrophy associated with MYO7A mutations in a large french cohort. Retina.

[30] Schultz J.M., Bhatti R., Madeo A.C. (2011). Allelic hierarchy of CDH23 mutations causing non-syndromic deafness DFNB12 or Usher syndrome USH1D in compound heterozygotes. J Med Genet.

[31] Bowne S.J., Daiger S.P., Hims M.M. (1999). Mutations in the RP1 gene causing autosomal dominant retinitis pigmentosa. Hum Mol Genet.

[32] Al-Rashed M., Abu Safieh L., Alkuraya H. (2012). RP1 and retinitis pigmentosa: report of novel mutations and insight into mutational mechanism. Br J Ophthalmol.

[33] Nanda A., McClements M.E., Clouston P. (2019). The location of exon 4 mutations in rp1 raises challenges for genetic counseling and gene therapy. Am J Ophthalmol.

[34] Bowne S.J., Sullivan L.S., Mortimer S.E. (2006). Spectrum and frequency of mutations in IMPDH1 associated with autosomal dominant retinitis pigmentosa and leber congenital amaurosis. Invest Ophthalmol Vis Sci.

[35] Aherne A., Kennan A., Kenna P.F. (2004). On the molecular pathology of neurodegeneration in IMPDH1-based retinitis pigmentosa. Hum Mol Genet.

[36] Naash M.L., Peachey N.S., Li Z.Y. (1996). Light-induced acceleration of photoreceptor degeneration in transgenic mice expressing mutant rhodopsin. Invest Ophthalmol Vis Sci.

[37] Tam B.M., Moritz O.L. (2009). The role of rhodopsin glycosylation in protein folding, trafficking, and light-sensitive retinal degeneration. J Neurosci.

[38] Tam B.M., Noorwez S.M., Kaushal S. (2014). Photoactivation-induced instability of rhodopsin mutants T4K and T17M in rod outer segments underlies retinal degeneration in X. laevis transgenic models of retinitis pigmentosa. J Neurosci.

[39] Iannaccone A., Man D., Waseem N. (2006). Retinitis pigmentosa associated with rhodopsin mutations: correlation between phenotypic variability and molecular effects. Vision Res.

