# Supplementary material for: Sector Retinitis Pigmentosa: Extending the Molecular Genetics Basis and Elucidating the Natural History
Source: Am J Ophthalmol. 2021 Jan;221:299–310. doi: 10.1016/j.ajo.2020.08.004 (PMC7772805; doi:10.1016/j.ajo.2020.08.004)
Supplement: Supplementary Figure 1 [file mmc1.docx]

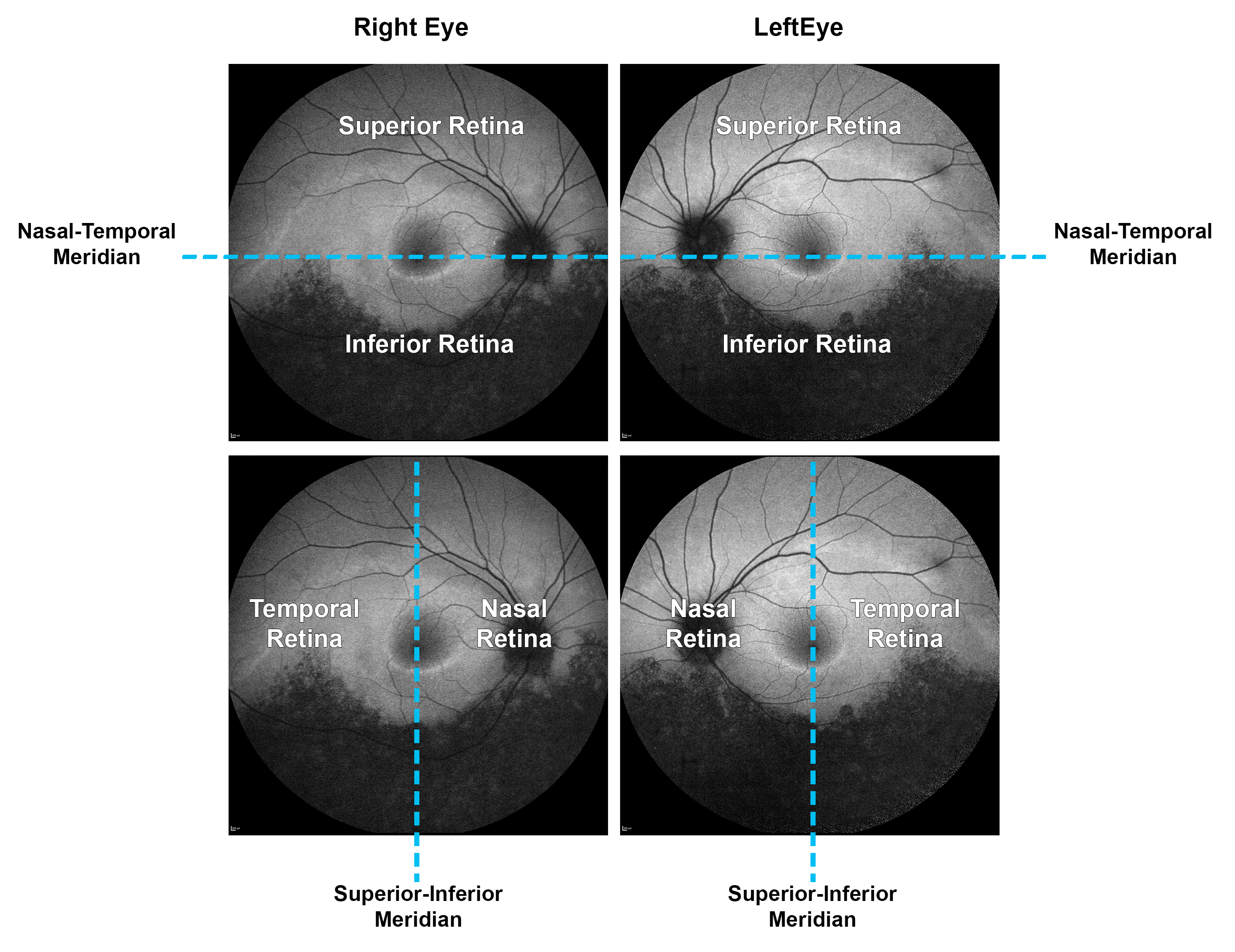


**Supplementary Figure 1: Example of Disease Localization**

The figure demonstrates how the retina was divided into four halves in a patient with sector retinitis pigmentosa (P26-*RHO*), both for right and left eye. The nasal-temporal meridian divided the retina into superior and inferior retina. The superior-inferior meridian divided the retina into nasal and temporal retina. In order for a half-retina to be considered affected; greater than 50% of the area needed to have decreased signal. In the presented example, the disease was localized to the inferior retina.
