# Supplementary material for: Sector Retinitis Pigmentosa: Extending the Molecular Genetics Basis and Elucidating the Natural History
Source: Am J Ophthalmol. 2021 Jan;221:299–310. doi: 10.1016/j.ajo.2020.08.004 (PMC7772805; doi:10.1016/j.ajo.2020.08.004)
Supplement: Supplementary Table 2 [file mmc3.doc]

| **Supplemental TABLE 2**. Fundus Autofluorescence Imaging Analysis | | | | | | | | | | | | | |
| --- | --- | --- | --- | --- | --- | --- | --- | --- | --- | --- | --- | --- | --- |
| Patient | Gene | Affected Retina at Baseline | | | | | Affected Retina at Follow-up | | | | | Follow-up | Progression |
| ID | Affected | Age  (y) | Superior | Inferior | Nasal | Temporal | Age  (y) | Superior | Inferior | Nasal | Temporal | Time  (y) |
| X-linked sector retinitis pigmentosa | | | | | | | | | | | | |  |
| P2*^ab^* | *RPGR* | 48.8 | NO | NO | YES | NO | 53.0 | NO | NO | YES | NO | 4.2 | YES |
| P3*^ab^* | *RPGR* | 28.7 | NO | YES | NO | YES | 35.4 | NO | YES | NO | YES | 6.7 | YES |
| P4*^ab^* | *RPGR* | 35.0 | NO | YES | NO | YES | 39.7 | NO | YES | NO | YES | 4.7 | NO |
| Autosomal recessive sector retinitis pigmentosa | | | | | | | | | | | | | |
| P7 | *MYO7A* | 57.4 | NO | YES | NO | NO | 62.4 | NO | YES | NO | NO | 5.0 | NO |
| P8*^ab^* | *MYO7A* | 54.1 | NO | YES | NO | NO | 59.4 | NO | YES | NO | NO | 5.3 | NO |
| P9*^b^* | *CDH23* | 18.3 | NO | YES | NO | NO | 24.4 | NO | YES | NO | NO | 6.1 | YES |
| P10*^ab^* | *EYS* | 56.0 | NO | YES | NO | YES | 60.0 | NO | YES | NO | YES | 4.0 | YES |
| Autosomal dominant sector retinitis pigmentosa | | | | | | | | | | | | | |
| P11*^ab^* | *IMPDH1* | 52.2 | NO | YES | NO | YES | 53.3 | NO | YES | NO | YES | 1.1 | NO |
| P12*^a^* | *IMPDH1* | 58.9 | NO | YES | NO | YES | 68.0 | NO | YES | NO | YES | 9.1 | NO |
| P13*^ab^* | *IMPDH1* | 70.4 | NO | YES | YES | YES | 74.8 | NO | YES | YES | YES | 4.4 | NO |
| P14*^ac^* | *RP1* | 53.8 | NO | YES | YES | YES | 59.6 | YES | YES | YES | YES | 5.8 | YES |
| P15*^ab^* | *RP1* | 26.0 | NO | YES | NO | YES | 30.4 | NO | YES | NO | YES | 4.4 | YES |
| P16 | *RP1* | 41.0 | NO | YES | NO | YES | 42.0 | NO | YES | NO | YES | 1.0 | NO |
| P18*^ab^* | *RHO* | 60.7 | NO | YES | NO | YES | 61.7 | NO | YES | NO | YES | 1.0 | NO |
| P21*^a^* | *RHO* | 38.2 | NO | YES | NO | YES | Not Available | | | | | Not Available | |
| P23*^ab^* | *RHO* | 45.6 | NO | YES | NO | NO | 52.6 | NO | YES | NO | NO | 7.0 | NO |
| P25*^ab^* | *RHO* | 53.5 | NO | YES | NO | NO | Not Available | | | | | Not Available | |
| P26*^abc^* | *RHO* | 30.3 | NO | YES | NO | NO | 38.0 | NO | YES | NO | NO | 7.7 | YES |

*^a^*Patients with peripapillary atrophy.

*^b^*Patients with hyperautofluorescent rim.

*^c^*Patients with perifoveal ring of increased signal.
