# Supplementary material for: Sector Retinitis Pigmentosa: Extending the Molecular Genetics Basis and Elucidating the Natural History
Source: Am J Ophthalmol. 2021 Jan;221:299–310. doi: 10.1016/j.ajo.2020.08.004 (PMC7772805; doi:10.1016/j.ajo.2020.08.004)
Supplement: Supplementary file 4 [file mmc2.zip › Supplementary_Table_revised.odt]

| Supplemental TABLE 1. Summary of Previously Reported Sequence Variants Causing Sector Retinitis Pigmentosa. |  |
| --- | --- |
| Gene | Nucleotide change | Amino acid Change/Effect | Reference(s) |  |
| Autosomal dominant sector RP |  |
| RHO | c.233A>T | p.Asn78Ile | Rivera-De la Parra et al.12 |  |
| RHO | c.44A>G | p.Asn15Ser | Kranich et al.11 |  |
| RHO | c.509C>A | p.Pro170His | Shah et al.16 |  |
| RHO | c.68C>A | p.Pro23His | Heckenlively et al.8 |  |
| RHO | c.316G>A | p.Gly106Arg | Fishman et al.9 |  |
| RHO | c.377G>T | p.Trp126Leu | Katagiri et al.40 |  |
| RHO | c.50C>T | p.Thr17Met | Fishman et al.9 |  |
| RHO | c.92T>A | p.Leu31Gln | Xiao et al.17 |  |
| RHO | c.544G>A | p. Gly182Ser | Fishman et al.9 |  |
| RHO | c.173C>G | p.Thr58Arg | Stone et al.6 |  |
| RHO | c.677T>C | p.Leu226Pro | Coussa et al.4 |  |
| RHO | c.173C>T | p.Thr58Met | Napier et al.15 |  |
| Autosomal recessive sector RP |  |
| SAG | Homozygous  c.926delA | p.Asn309Thrfs*12 | Nakamachi et al.20 |  |
| CDH23 | c.8530C>A, c.5820+5G>A | p.Pro2844Thr, p.? | Branson et al.18 |  |
| RDH5 | Homozygous c.319G>C | p.Gly107Arg | Sato et al.19 |  |
| USH1C | c.308G>A, c.2227-1G>T | p.Arg103His | Saihan et al.2 |  |
| X-Linked sector RP |  |
| RPGR | c.3092delA | p.Glu1031Glyfs*58 | Nguyen et al.21 |  |
| RPGR | c.3092_3093delAG | p.Glu1031Glyfs*47 | Coussa et al.4 |
| RP = retinitis pigmentosa. Gene (transcript GenBank accession ID): CDH23 (NM_022124); RDH5 (NM_002905.5); RHO (NM_000539); RPGR (NM_001034853); SAG (NM_000541.5); USH1C (NM_153676). Disease-causing variants are subdivided based on mode of inheritance. The causative gene is specified along with the corresponding c.DNA and protein changes as reported in the studies, along with the specific reference(s).  For the autosomal recessive alleles, variants are separated by a comma. |

Reference

40. Katagiri S., Hayashi T., Akahori M., et al. RHO Mutations (p.W126L and p.A346P) in  Two Japanese Families with Autosomal Dominant Retinitis Pigmentosa. J Ophthalmol.  2014;2014(210947).
